# Docosahexaenoic acid in ARSACS: observations in two patients

**DOI:** 10.1186/s12883-020-01803-3

**Published:** 2020-05-28

**Authors:** Ivana Ricca, Alessandra Tessa, Rosanna Trovato, Giacomo Maria Bacci, Filippo Maria Santorelli

**Affiliations:** 1 Molecular Medicine, IRCCS Fondazione Stella Maris, via dei Giacinti 2- 56128 Calambrone-, Pisa, Italy; 2 Pediatric Ophthalmology Unit, Meyer Children’s Hospital, University of Florence, Florence, Italy

**Keywords:** ARSACS, Sacsin, 6-min-walking test (6MWT)

## Abstract

**Background:**

Spastic ataxia of Charlevoix-Saguenay is a neurodegenerative condition due to mutations in the *SACS* gene and without a cure. Attempts to treatments are scarce and limited to symptomatic drugs.

**Case presentation:**

Two siblings harboring biallelic variants in *SACS* underwent oral supplementation (600 mg/die) with docosahexaenoic acid (DHA), a well-tolerated dietary supplement currently used in SCA38 patients. We assessed over a 20 month-period clinical progression using disease-specific rating scales.

**Conclusions:**

DHA was safe over a long period and well-tolerated by the two patients; both showed a stabilization of clinical symptoms, rather than the expected deterioration, warranting additional investigations in patients with mutations in *SACS*.

## Background

Autosomal recessive spastic ataxia of Charlevoix-Saguenay (ARSACS) is a relatively common early-onset inherited disorder characterized by the combination of cerebellar ataxia, pyramidal signs, peripheral neuropathy and retinal involvement [1]. ARSACS is due to mutations in *SACS*, which encodes sacsin, a molecular chaperone involved in protein quality control, mitochondrial network dynamics and neurofilament homeostasis. The pathological mechanisms underlying neurodegeneration in sacsin-mutated neurons are not fully understood. A recent study in neuronal models of sacsin depletion suggests that loss of the protein could selectively impair mitophagy, leading to accumulation of damaged mitochondria and subsequent bioenergetic dysfunction, increased levels of oxidative stress products, and peroxidation of membrane phospholipids [2]. The hypothesis of bioenergetic and autophagy dysfunction in ARSACS pathogenesis suggests a potential therapeutic role for agents targeting these pathways.

Docosahexaenoic acid (DHA) is a well-tolerated dietary supplement able to cross the blood-brain barrier. It is the main component of brain phospholipids and is essential for normal central nervous system development. DHA derives mainly from diet and liver metabolism, while production within the brain itself is limited [3]. DHA has several neurobiological effects. It has neuroprotective properties, promoting neuronal survival and repair through neurotrophic, antiapoptotic and anti-inflammatory signaling [4]. By blocking mTOR signaling, DHA also stimulates the autophagic pathway [5]. It is used in other neurodegenerative disorders characterized by cerebellar and pyramidal tract involvement, such as in patients with spinocerebellar ataxia 38 [6, 7] and in mouse models of Friedreich’s ataxia [8]. DHA is usually safe and well-tolerated, though adverse effects include gastrointestinal discomfort and diarrhea.

## Case presentation

We here report clinical observations in two siblings with ARSACS who underwent DHA oral supplementation, 600 mg per day, for 20 months. Their full clinical and molecular data have been described elsewhere [9]. Briefly, P1 is a 41-year-old woman affected by ataxic-spastic signs, evident from the age of 15 years. Her clinical evaluation before starting DHA supplementation (T0, 39 years) showed moderate spastic-ataxic gait, mild dysarthria, mild diffuse leg weakness and dysmetria, bilateral Babinski sign, *pes cavus* and hammertoes. She scored 16/52 and 7/40 on the Spastic Paraplegia Rating Scale (SPRS) [10] and the Scale for the Assessment and Rating of Ataxia (SARA) [11], respectively. Her disease-specific severity index (DSI-ARSACS) [12] score was 11/32. She completed the 10-m walking test (10MWT) [13] in 15.8 s and covered a distance of 252 m on the 6-min walking test (6MWT) [13]. Electroneurography showed severe sensorimotor axonal and demyelinating polyneuropathy. Brain MRI documented mild cerebellar atrophy, prominent in the cerebellar vermis, and hypointense stripes in the pons. Spine MRI was normal. Her 40-year-old brother (case P2) displayed a similar neurological phenotype, present from the age of 16. Before starting DHA, his SPRS, SARA and DSI-ARSACS scores were 12/52, 9/40 and 9/32, respectively. He performed the 10MWT in 9.9 s and walked 400 m on the 6MWT. MRI showed slight atrophy of the superior cerebellar vermis, corpus callosum thinning, and cervical spinal cord atrophy. Neurophysiological examinations were consistent with sensorimotor axonal polyneuropathy.

**Fig. 1:**
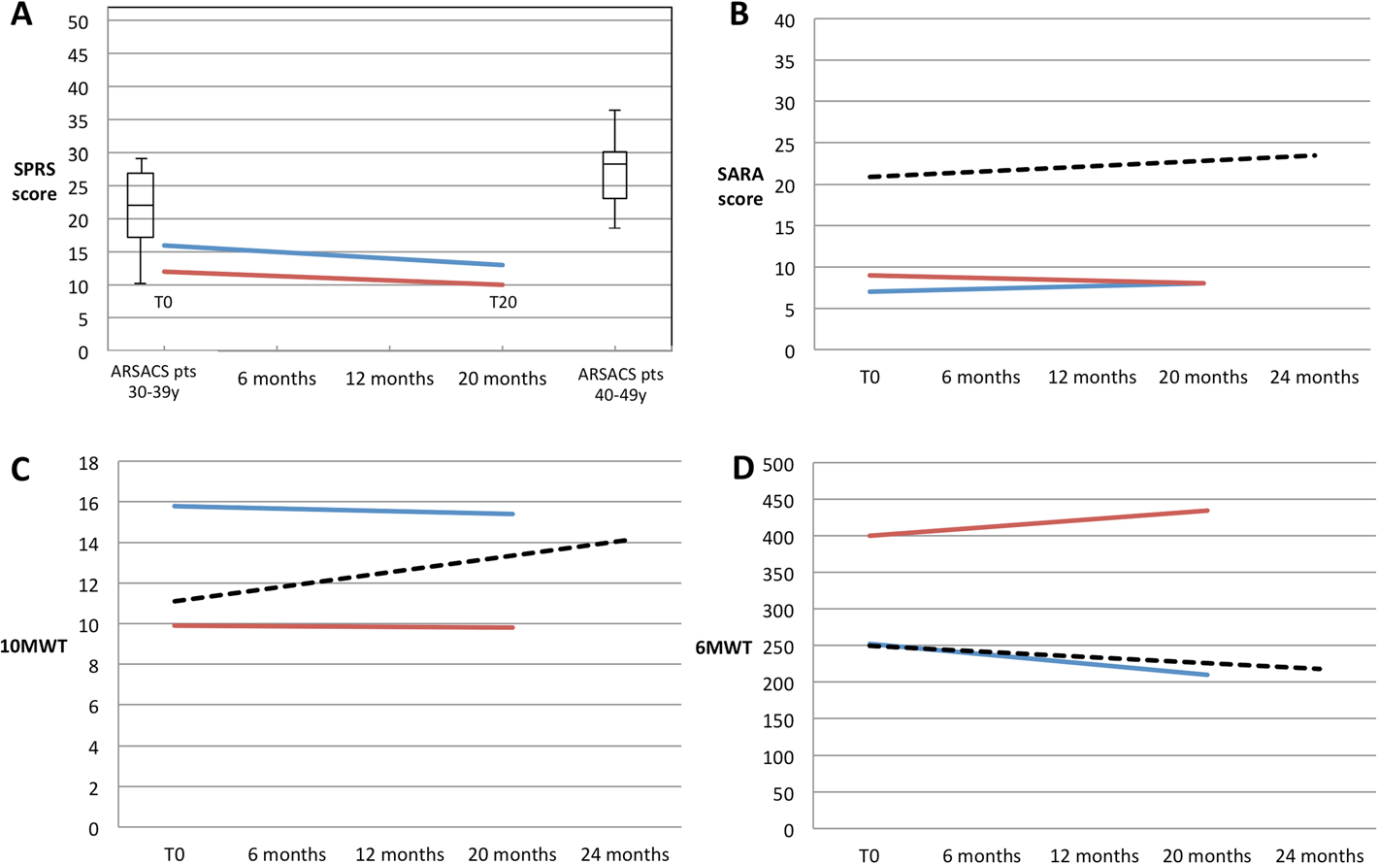
Functional evaluations during DHA therapy. The figure summarizes trends of SPRS, SARA, 10MWT and 6MWT scores in P1 and P2 before starting DHA (T0) and after 20 months of treatment. The SPRS (**a**) and SARA (**b**) scores, time taken to perform the 10MWT (seconds) (**c**), and meters covered on the 6MWT (**d**) are reported for both patients (P1, blue lines; P2, red lines). The box-plots in (**a**) display the SPRS scores of ARSACS patients in the age groups 30–39 and 40–49 (derived from [15]). The dashed lines in (**b**), (**c**) and (**d**) represent the motor performance trends reported in the literature in aged-matched ARSACS patients with spastic-ataxic diseases measured by SARA, 10MWT and 6MWT (reported in [14])

Over the 20-month period, DHA was safe and well-tolerated by the two patients; both showed an overall stabilization of clinical symptoms, as measured by SARA, SPRS, DSI-ARSACS, 6MWT and 10MWT (Table 1; Fig. 1), rather than the expected deterioration [14, 15]. After DHA supplementation, P1 had SPRS, SARA, and DSI-ARSACS scores of 13/52, 8/40 and 9.5/32, respectively. She showed a mild improvement in lower limbs functions (DSI-ARSACS items 5–7) and a mild deterioration of the upper right limb dexterity (SARA items 5 and 7; DSI-ARSACS item 3). She performed the 10MWT in 15.4 s and walked 250 m on the 6MWT. Her brother’s scores were SPRS 10/52, SARA 8/40, and DSI-ARSACS 9.5/52. He showed mild improvement in speech (SARA item 4; DSI-ARSACS item 1) and in lower limb functions (DSI-ARSACS items 5 and 7) and a mild deterioration of right limbs dexterity (SARA items 5 and 8; DSI-ARSACS item 3). He did the 10MWT in 9.8 s and walked 434.5 m on the 6MWT. In both P1 and P2 the health-related quality of life scores assessed through the EQ-5D-3 L [16] questionnaire were unchanged. However, at 20 months of therapy, serum low-density lipoprotein (LDL) and total cholesterol levels in P1 were found, by chance, to be raised (241 [normal < 100] and 282 mg/dL [normal < 200], respectively). These findings may indicate familial hypercholesterolemia (FH). Indeed, the patients’ neurologically-healthy father had died at the age of 40 of myocardial infarction linked to severe hypercholesterolemia. Reviewing blood tests previously performed in P1, we noticed that her basal LDL and total cholesterol levels were already high at T0 (177 and 241 mg/dL, respectively). HDL cholesterol levels were in the normal range at T0 (50 mg/dL) and low after 20 months (30 mg/dL [normal > 40). Triglycerides levels were always in the normal range (62 mg/dL at T0 and 54 mg/dL after 20 months [normal < 150]). Informed of the cardiovascular risks related to dyslipidaemia, because of lack of sufficient data about the safety of DHA administration in subjects with LDL cholesterol elevation and/or FH, both patients chose to interrupt treatment.

**Table 1:** SPRS, SARA, DSI-ARSACS, 10MWT and 6MWT scores in patients 1 and 2 before starting DHA and after 6, 12 and 20 months of DHA treatment

	P1				P2			
	T0	6 mo	12 mo	20 mo	T0	6 mo	12 mo	20 mo
SPRS	16	17	15.5	13	12	9.5	10	10
SARA	7	8.75	11.5	8	9	7.5	11	8
DSI-ARSACS	11	9.5	7.5	9.5	9	9.5	8.5	9.5
10MWT (sec)	15.8	14.2	13.8	15.4	9.9	9.2	9.6	9.8
6MWT (m)	252	253	264	250	400	475	434	434.5

## Discussion and conclusion

Description of pharmacological management in rare neurodegenerative conditions is useful to advice patients in case of self-administration and use of over-the-counter drugs, especially when no cures are seen over the horizon [17]. The reported data suggest that DHA supplementation in ARSACS patients seems to be safe and well tolerated and a promising add-on therapy in the complex treatment of this condition. Nevertheless, our anecdotal report should be read in view of future investigations in larger groups of subjects also to confirm long-term safety.

DHA supplementation is associated with modifications in lipids profile and some studies suggest that DHA down-regulates expression of LDL receptors and LDL-cholesterol clearance [18]. Therefore during DHA administration, lipoprotein pools in plasma should be monitored, especially in subjects with FH.

## Data Availability

All data generated or analyzed during this study are included in this published article.

## Abbreviations

SPRS: Spastic Paraplegia Rating Scale
SARA: Scale for the Assessment and Rating of Ataxia
DSI-ARSACS: Disease-specific severity index for adults with autosomal recessive spastic ataxia of Charlevoix-Saguenay (ARSACS)
10MWT: 10-m-walking test
6MWT: 6-min-walking test
P1 / P2: Patient 1 / patient 2
DHA: Docosahexaenoic acid
T0: Before treatment;
6 mo / 12 mo / 20 mo: After 6 / 12 / 20 months of treatment
